# *PARP1* expression and its correlation with survival is tumour molecular subtype dependent in glioblastoma

**DOI:** 10.18632/oncotarget.18013

**Published:** 2017-05-19

**Authors:** Balázs Murnyák, Mahan C. Kouhsari, Rotem Hershkovitch, Bernadette Kálmán, György Marko-Varga, Álmos Klekner, Tibor Hortobágyi

**Affiliations:** ^1^ Division of Neuropathology, Institute of Pathology, Faculty of Medicine, University of Debrecen, Debrecen, Hungary; ^2^ Institute of Diagnostics, Faculty of the Health Sciences, University of Pecs, Pecs, Hungary; ^3^ Molecular Pathology Unit, Markusovszky Teaching Hospital, Szombathely, Hungary; ^4^ Division of Clinical Protein Science & Imaging, Department of Biomedical Engineering, Lund University, Lund, Sweden; ^5^ Department of Neurosurgery, Faculty of Medicine, University of Debrecen, Debrecen, Hungary; ^6^ Institute of Psychiatry, Psychology and Neuroscience, King's College London, London, UK

**Keywords:** glioma, glioblastoma, p53, PARP1

## Abstract

Overexpression of PARP1 exists in various cancers, including glioblastoma (GBM). Although PARP1 inhibition is a promising therapeutic target, no comprehensive study has addressed PARP1's expression characteristics and prognostic role regarding molecular heterogeneity in astrocytomas including GBM. Our aim was to evaluate PARP1's associations with survival, WHO grade, lineage specific markers, and GBM transcriptomic subtypes. We collected genomic and clinical data from the latest glioma datasets of The Cancer Genome Atlas and performed PARP1, ATRX, IDH1, and p53 immunohistochemistry on GBM tissue samples. We demonstrated that PARP1 gain and increased mRNA expression are characteristics of high-grade astrocytomas, particularly of Proneural and Classical GBM subtypes. Additionally, higher *PARP1* levels exhibited an inverse correlation with patient survival (p<0.005) in the Classical subgroup. *ATRX* (p=0.006), and *TP53* (p=0.015) mutations were associated with increased PARP1 expression and PARP1 protein level correlated with *ATRX* loss and p53 overexpression. Furthermore, higher *PARP1* expression together with wildtype *TP53* indicated shorter survival (p=0.039). Therefore, due to subtype specificity, *PARP1* expression level and *TP53* mutation status are reliable marker candidates to distinguish Proneural and Classical subtypes, with prognostic and therapeutic implications in GBM.

## INTRODUCTION

Glioblastoma (GBM) is a highly aggressive and the most prevalent primary brain tumour in adults. GBM has a dismal outcome, the median survival is typically less than 2 years [1]. The current treatment consists of maximal surgical resection of the tumour followed by concurrent chemo- (TMZ, temozolomide) and radiation therapy (RT) [2]. Despite extensive efforts to improve treatment, GBM is still resistant to current postoperative therapies making GBM a compelling field of cancer research [3]. Improving the outcome requires novel diagnostic approaches and effective treatment strategies [4].

The biological basis of the resistance involves many factors, including molecular heterogeneity, and impaired DNA repair mechanisms. Currently, targeting the poly(ADP-ribose) polymerase 1 (PARP1) DNA repair protein in GBM is a new and promising aspect in clinical trials [5]. PARP1 is a nuclear protein, and is normally involved in DNA repair and the maintenance of genomic stability [6]. PARP1 binds to DNA strand breaks and produces a poly (ADP-ribose) chain from NAD^+^ substrate which signals the cell to initiate DNA damage repair. The role of PARP1 has been already investigated in brain diseases [7, 8], and its overexpression has been reported in various tumour types, such as breast, ovary, skin, colorectum, lung and brain [9–11]. Upregulated PARP1 can enhance the anti-apoptotic property of tumours resulting in resistance to DNA damaging therapeutic agents [12]. PARP inhibitors (PARPi) sensitize tumour cells to radiotherapy and to chemotherapeutic agents. The inhibitors attach to the catalytic domain of the protein thereby blocking synthesis of ADP-ribose polymers. Consequently, the cellular responses to DNA damage do not occur, leading to cell death [6].

Although all GBMs share common histological features, they individually vary at their molecular level resulting in significant differences in terms of prognosis and response to treatment [13]. In the last decade, numerous high-scale genomic profiling studies were performed to elucidate the biology of GBM. In addition, large scale functional proteomics studies were performed, where ENCODE software developments were used to integrate RNA-Seq and its correlation to protein expression, and regulation in GBM patients [14, 15]. The proteogenomic initiative runs as a HUPO (www.HUPO.org) disease initiative, recently reported on [16]. In a comprehensive analysis by The Cancer Genome Atlas (TCGA) project, dysregulation of p53, Rb and receptor tyrosine kinase pathways were identified [17]. Recently, several glioma lineage and GBM subtype specific molecular alterations were described, indicating a diversity amid similar histological types. The identification of isocitrate dehydrogenase 1 and 2 (*IDH1* and *IDH2*) mutations was a breakthrough in the field of GBM research. Soon after it became an important diagnostic and prognostic marker in gliomas [18]. To date, GBM can be divided into IDH-wildtype and mutant based on their IDH status [19]. Furthermore, *IDH1* together with Alpha Thalassemia/Mental Retardation Syndrome X-linked (*ATRX*) and *TP53* status are reliable diagnostic and prognostic markers for the astrocytoma lineage and they are also relevant in GBM stratification. Beyond that, GBM was further divided into four molecular subtypes: Classical (CL), Mesenchymal (ME), Proneural (PN) and Neural (NE), based on their chromosomal structural alterations, copy number alterations (CNAs), point mutation status and gene expression profiles [20–22].

**Figure 1:**
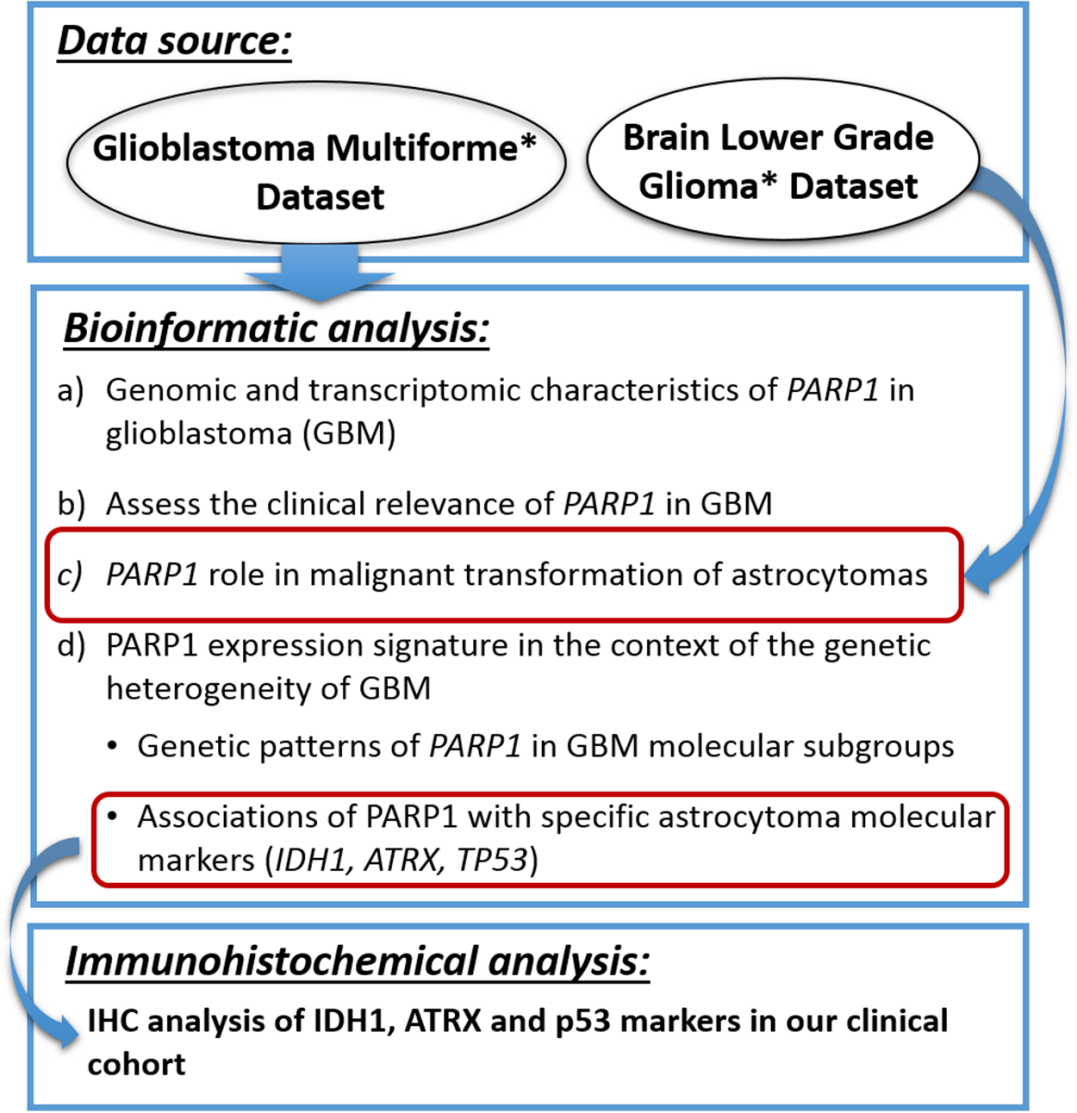
The overview of the analysis strategy for *PARP1* characterisation used in this study

Although the efficiency of PARP1 inhibition was already established in GBM, its molecular characteristics and prognostic role regarding molecular heterogeneity is not fully understood. The aim of our study is to analyse the role of PARP1 using the latest genomic datasets of the TCGA, and an independent clinical cohort (Figure 1). To the best of our knowledge, we hereby present the first comprehensive study that evaluate I) *PARP1* CNAs and mRNA expressions, II) its usefulness in GBM subtype prognosis, and III) the associations of *PARP1* with specific astrocytoma molecular markers. Finally, IV) we demonstrate a close link between *PARP1* and *TP53* that can serve as a prognostic and diagnostic marker for GBM.

## RESULTS

### Genomic and transcriptomic characteristics of PARP1 in GBM

We first analysed *PARP1* mutations and copy numbers of GBM specimens in the TCGA database via cBioPortal (http://www.cbioportal.org/public-portal/). Only two *PARP1* somatic mutations were observed (V948I and A709T) in GBM. Information on CNA data was available for 562 GBM samples. *PARP1* exhibited a low-level gain and heterozygous deletions in more than 14% and 6% of the cases, respectively. On the other hand, *PARP1* amplification was infrequent (0.35%), however homozygous deletion was not observed.

Next, we examined the relative *PARP1* mRNA levels in GBM. The database included 135 adult GBM cases with DNA sequencing, CNAs and mRNA expression data according to the screening criteria detailed in Materials and Methods. In addition, an increased expression was observed (z-score ≥1) in 20.74% (28/135) of the cases.

**Figure 2:**
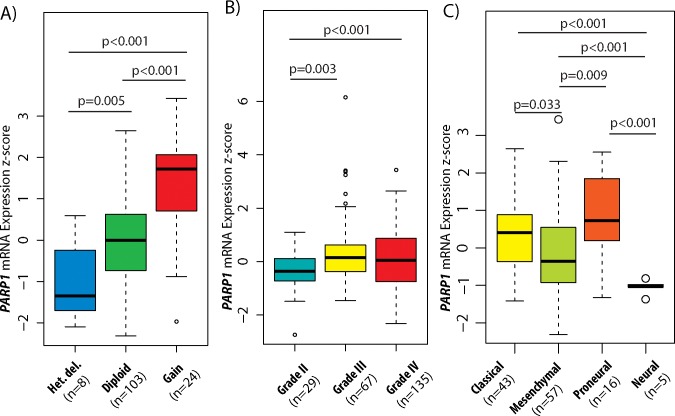
PARP1 copy number and expression levels in GBM subtypes and glioma WHO grades Association between copy number alterations (CNAs) and mRNA expressions of *PARP1* in GBM **(A)**. *PARP1* mRNA expression level z-scores in different grades of astrocytomas **(B)** and in GBM subtypes **(C)**. The TCGA glioblastoma multiforme (WHO grade IV) & brain lower grade glioma (WHO grade II & III) TCGA datasets were used for the analysis.

Lastly, for determining the possible regulation mechanisms associated with *PARP1* expression values, we evaluated the correlation between CNAs and mRNA levels. A significant expression difference was found between heterozygous deletion and diploid (p=0.005), gain and diploid (p<0.001), as well as heterozygous deletion and gain statuses (p<0.001). These results demonstrate a close link between the copy number and gene expression (Figure 2A).

### *PARP1* levels are increased in higher grade astrocytomas

To examine the significance of *PARP1* in malignant transformation of astrocytomas, the Brain Lower Grade Glioma (TCGA, Provisional) dataset was investigated through cBioPortal. Altogether, CNA data of 169 lower grade tumours (55 grade II and 114 grade III astrocytomas) were added to the 562 GBM samples. We found, that grade II tumours have the lowest CNA rate (0.036) followed by grade III astrocytomas (0.167) and grade IV GBM (0.215), and high *PARP1* copy numbers correlate with higher histological grades (p<0.001) (Supplementary Figure 1). Homozygous deletions occurred in only 0.1% of the grade II tumours. *PARP1* amplification was also rare, found in only 0.3% of GBMs. In total, mRNA expression data were available for 97 lower-grade astrocytomas (29 grade II and 67 grade III tumours). Increased *PARP1* mRNA levels were found in the high-grade tumours as opposed to grade II astrocytomas (GII vs. GIII, p=0.003, and GII vs. GIV, p<0.001). Although *PARP1* expression was increased in both grade III and IV tumours, there were no significant changes between those two grades (Figure 2B). *PARP1* CNAs showed significant differences in distribution among WHO grades (p<0.001).

### Mutation status of *ATRX* and *TP53* genes are associated with *PARP1* expressions

**Figure 3:**
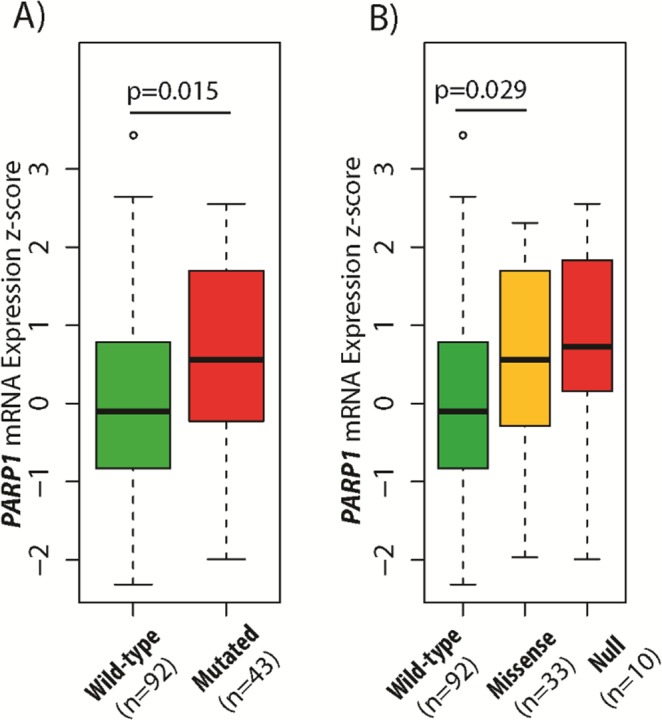
Association between *TP53* gene status and *PARP1* mRNA expression in glioblastoma *PARP1* mRNA expression was higher in mutated *TP53* cases **(A)**, but only missense *TP53* mutations showed significant difference compared to wild-type *TP53* **(B)**. The TCGA glioblastoma multiforme, provisional dataset was used for the analysis.

We evaluated the *PARP1* expression signature in the context of the genetic heterogeneity of GBM. We analysed the association between *PARP1* levels and the mutation status of astrocytoma lineage specific genes such as *IDH1*, *ATRX* and *TP53*. We discovered that samples carrying mutated *ATRX* (p=0.006) and *TP53* (p=0.015) were associated with higher *PARP1* expression levels (Table 1). The IDH status of GBMs did not show any association with PARP1 expression. The TCGA dataset contains only the IDH and *ATRX* status of the tumours (Table 2). To examine the association between *PARP1* and *TP53* status, cases were classified into wildtype (n=92) and mutant *TP53* (n=43) tumours. Next, mutant *TP53* samples were further divided into missense (n=33) and null (n=10) mutations in order to investigate the mutation effects on *PARP1* expressions (Figure 3A, Table 2). *PARP1* mRNA expression was higher in *TP53* mutated cases (p=0.015) than in its wildtype counterpart. Importantly, there was no significant changes between missense and null *TP53* mutations (Figure 3B).

**Table 1:** Correlations between the mRNA expressions of *PARP1* and key molecular glioma markers

Key glioma markers	N	Meanz-score	± SE^1^	p-Value	95% CI^2^	
					Lower	Upper
***ATRX* status**						
Wild-type	128	0.130	0.103	***0.006***	−2.124	−0.367
Mutant	7	1.375	0.271			
***IDH* status**						
Wild-type	127	0.158	0.104	0.151	−1.457	0.228
Mutant	8	0.772	0.369			
***TP53* status**						
Wild-type	92	0.028	0.118	***0.015***	−0.943	−0.101
Mutant	43	0.550	0.182			

(Significant p-values are marked in bold.)

^1^Standard error; ^2^confidence of interval.

**Table 2:** Patient characteristics with available sequencing, CNA, and mRNA expression data

	Brain lower grade glioma (TCGA, provisional) (n=97)	Glioblastoma multiforme (TCGA, provisional) (n=135)
**Diagnosis age (years)**		
	42.31 ± 13.14	60.59±13.48
	(range: 20-74)	(range: 21-89)
**Overall survival (months)**		
	24.76 ± 22.17	13.33 ± 12.64
**Sex**		
Female	42	45
Male	55	90
**WHO grade**^1^		
II	29	-
III	67	-
IV	-	135
***ATRX* status**		
Mutant	45	7
Wild-type	52	128
***IDH* status**		
Mutant	66	8
Wild-type	31	127
***TP53* status**		
Missense mut.	51	33
Null mut.	14	10
Wild-type	32	92

^1^WHO grade was unknown in one sample.

### Immunohistochemical expression of PARP1 is correlated with ATRX and p53

**Figure 4:**
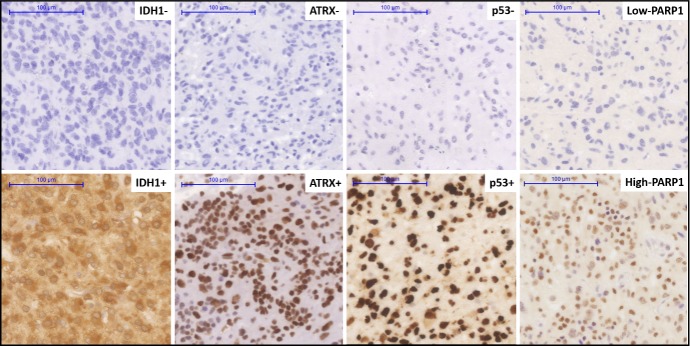
Immunohistochemical staining pattern for various markers in a clinical cohort of glioblastoma Immunohistochemical positive and negative staining for IDH1^R132H^, positive and negative staining patterns of ATRX and p53, high and low nuclear expression of the PARP1. Original magnification: x200. Scale bar: 100 μm.

We immunohistochemically studied PARP1 in a series of GBMs to evaluate its potential differential expression in association with key markers at protein levels. PARP1 staining was primarily localized in the nucleus of tumour cells, consistent with previous studies [11] (Figure 4). Ninety percent (54/60) of all cases were PARP1 positive, while 10% (6/60) were negative. The relationships between PARP1 and ATRX, IDH1 and p53, respectively, were also evaluated. In our clinical cohort, PARP1 IHC expression was significantly associated with the expression of p53 (p=0.0281) and ATRX (p=0.002) but not with that of IDH1 (Table 3).

**Table 3:** Immunohistochemical expression of PARP1 and its relationship with key molecular glioma markers

	PARP1 IHC expression		p-Value
	Negative	Positive	
**Sex**			
Female	2	28	0.3894
Male	4	26	
**Age**			
Mean ± SD*	61.69 ± 6.45	58.52 ± 9.18	0.415
**p53 expression**			
Negative	4	13	***0.0281***
Positive	2	41	
**IDH1 expression**			
Negative	6	52	0.6316
Positive	0	2	
**ATRX expression**			
Negative	3	4	***0.0020***
Positive	3	50	

(Significant p-values are marked in bold).

*Standard deviation.

### *PARP1* mRNA levels are increased in PN and CL GBM subtypes

In the exploration of the subtype-specific role of *PARP1* in GBM, we identified that PN and CL subtypes showed an increased *PARP1* expression (Figure 2C). More specifically, PN (mean z-score: 0.801) and CL (mean z-score: 0.375) subtypes have the highest average *PARP1* mRNA level, followed by ME (mean z-score: -0.121) and NE (mean z-score: -1.049), respectively.

Simultaneously, we also assessed whether *PARP1* CNAs were related to specific GBM subtypes. CNAs information on transcriptional subtypes were available for 475 cases. We found that the PN subtype had the highest CNA rate (0.275), followed by the ME (0.226), NE (0.224), and CL (0.129) subtypes. *PARP1* copy number gains were observed in all subtypes: ME (4.4%), PN (4.4%), CL (2.9%) and NE (2.3%). Heterozygous deletion of *PARP1* was found in 3.4% of ME GBMs. PARP1 amplification occurred only in CL and ME subtypes (0.2-0.2%) (Supplementary Figure 2).

### High PARP1 expression is associated with shorter survival in CL GBM subtype

**Figure 5:**
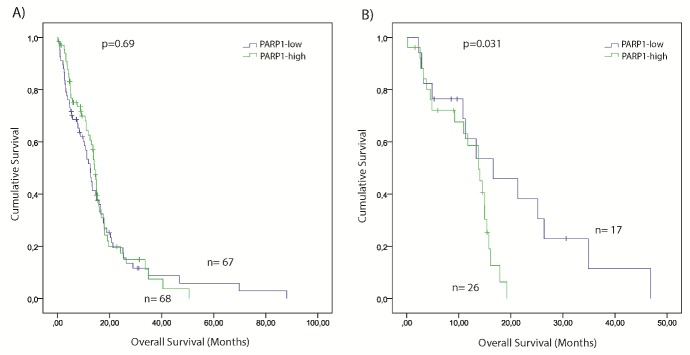
Kaplan–Meier plot of overall survival (OS) stratified by high and low *PARP1* mRNA expression in GBM (A) and in Classical GBM subgroup (B). The TCGA glioblastoma multiforme, provisional dataset was used for the analysis. (Significance was assessed by the log-rank method.)

To assess the clinical relevance of *PARP1* in GBM, we examined the association between *PARP1* mRNA expression and the overall survival in TCGA GBM samples. The mean overall survival (OS) of the TCGA patients was 13.3 months. GBM cases were assigned into PARP1-high (n=68) and PARP1-low (n=67) groups, using median *PARP1* mRNA expression z-score as cut off value. Although patients with PARP1-low expression had longer survival, there were no statistically significant differences observed between the groups (Figure 5A).

The mean OS of TCGA patients across different GBM subtypes was the following: CL=12.7; PN=19.8; ME=11.7, and NE=7.7 months. Kaplan-Meier survival analysis indicated that patients of the PARP1-low group had statistically significant shorter overall survival time compared with their PARP1-high counterpart (p=0.031) (Figure 5B). No significant differences were noted in the survival values between the PARP1-low, and -high expression cohorts of other GBM subtypes.

We also investigated whether *PARP1*'s genetic signatures were related to the clinicopathological characteristics of GBM patients. No significant associations were observed between *PARP1* status and age and sex (Table 3).

### High *PARP1* level together with wildtype *TP53* predict a shorter survival in GBM

**Figure 6:**
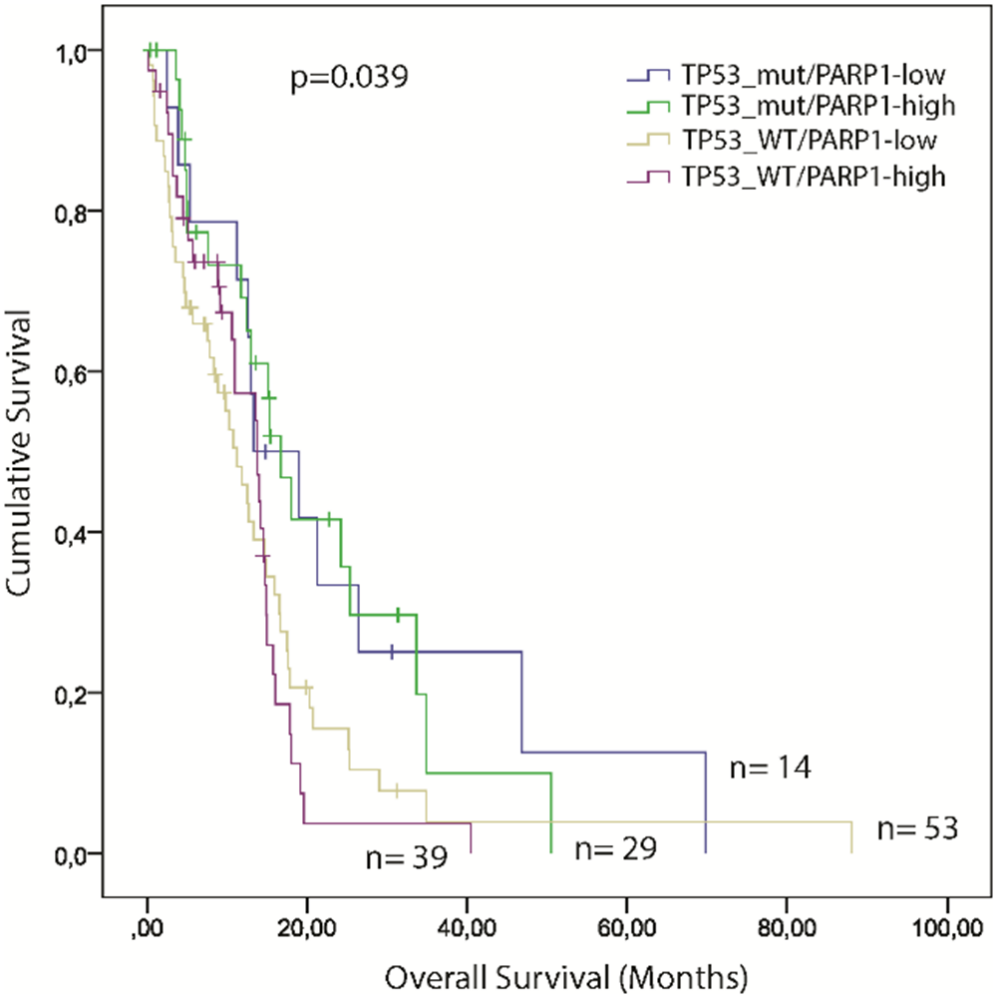
Kaplan–Meier plot of overall survival (OS) stratified by the combination of high and low *PARP1* mRNA expression and *TP53* mutation status in GBM. The TCGA glioblastoma multiforme, provisional dataset was used for the analysis. (Significance was assessed by the log-rank method.)

We analysed cumulative effects of p53 and PARP1 on survival with GBM. TCGA samples were further classified according to their *PARP1* levels and *TP53* mutation status: I) *TP53*^mut^/PARP1-high (n=29); II) *TP53*^mut^/PARP1-low (n=14); III) *TP53*^WT^/PARP1-high (n=39); and IV) *TP53*^WT^/PARP1-low (n=53). We found, that GBM patients with wild-type *TP53* gene and PARP1-high level have a shorter survival (p=0.039) compared to the other groups (Figure 6).

### *PARP1* status is associated with the p53 pathway

**Figure 7:**
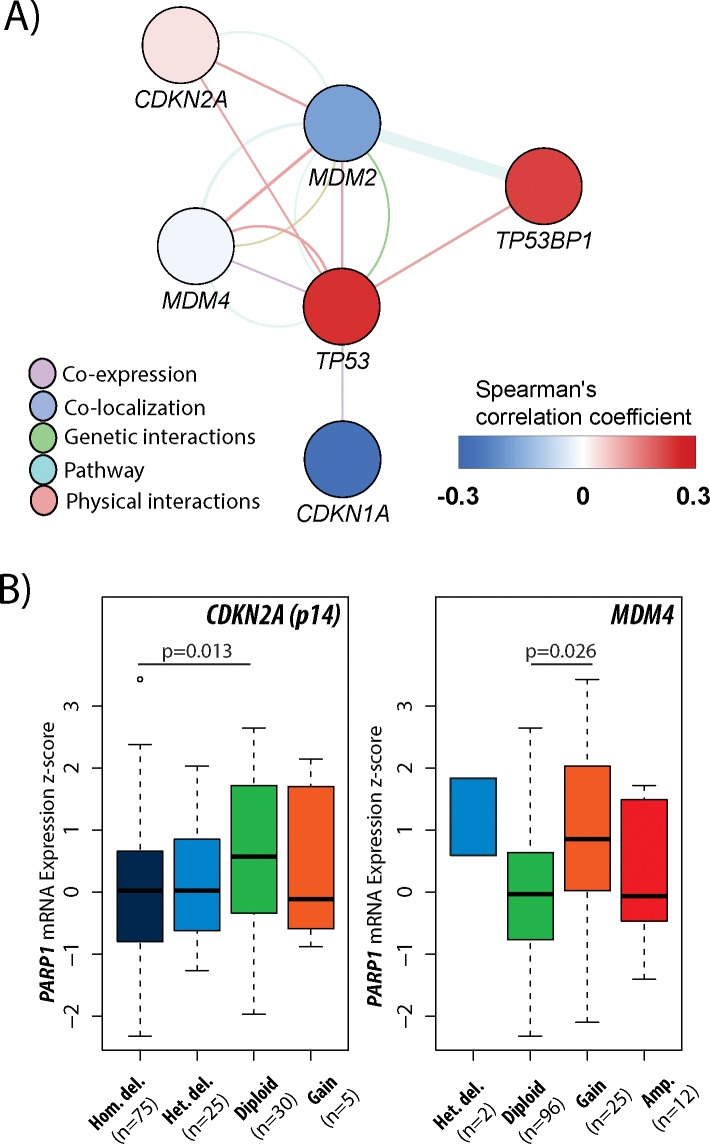
Association between *PARP1* and p53 pathway in glioblastoma A gene-gene interaction network presenting the correlation between PARP1 and the general members of p53 pathway in GBM according to mRNA expressions **(A)**. The associations of *PARP1* mRNA expression levels with *CDKN2A* and *MDM4* copy number alterations in GBM **(B)**. The TCGA glioblastoma multiforme, provisional dataset was used for the analysis.

**Figure 8:**
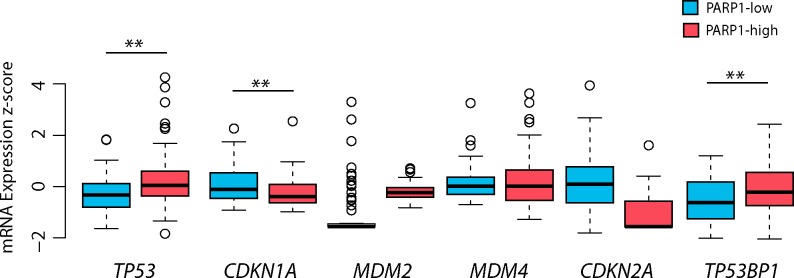
The impact of *PARP1* levels on p53 pathway-related genes in glioblastoma The mRNA expression of *TP53* and *TP53BP1* was increased in PARP1-high group, whereas *CDKN1A* (p21) mRNA expression was higher in PARP1-low group. The TCGA glioblastoma multiforme, provisional dataset was used for the analysis. Level of significance: * (p < 0.05), ** (p < 0.01), and *** (p < 0.001).

To extend our investigations, the relationship between *PARP1* status and the p53 pathway (*CDKN1A* (p21), *MDM2*, *MDM4*, *CDKN2A* (p14), and *TP53BP1*) was also examined. Regarding mRNA expressions, PARP1 displayed significant correlation with *TP53, CDKN1A*, and *TP53BP1* (Table 4, Figure 7A). Accordingly, mRNA expression levels of *TP53* (p=0.003), and *TP53BP1* (p=0.004) were increased in PARP1-high, and *CDKN1A* (p=0.009) was increased in PARP1-low GBMs (Table 5, Figure 8). *PARP1* levels showed correlation with copy number alterations of *CDKN2A* and *MDM4* genes (Figure 7B). More specifically, *PARP1* levels were decreased in homozygously deleted *CDKN2A* (p=0.013) cases, and increased in cases with *MDM4* gain (p=0.026) when we compared CNAs to the diploid variants of the genes. However, there was no significant association between *MDM2* CNAs and *PARP1*.

**Table 4:** Relationship between mRNA expressions of *PARP1* and components of the p53 pathway

Genes	Kendall's tau b		Spearman's rho	
	Cor. coef.	p-Value	Cor. coef.	p-Value
***CDKN1A***	−0.187	***0.001***	−0.272	***0.001***
*CDKN2A*	0.028	0.629	0.047	0.587
*MDM2*	−0.111	0.057	−0.168	0.051
*MDM4*	−0.003	0.953	−0.004	0.961
***TP53***	0.169	***0.004***	0.246	***0.004***
***TP53BP1***	0.162	***0.005***	0.230	***0.007***

**Table 5:** The impact of PARP1-low and high mRNA expression levels in the p53 pathway

Genes	PARP1 levels^1^		p-Value	± SE^2^	95% CI^3^	
	Low	High			Lower	Upper
***CDKN1A***	0.075	−0.227	***0.009***	0.113	0.077	0.526
*CDKN2A*	−1.117	−1.029	0.560	0.151	−0.387	0.210
*MDM2*	2.363	5.240	0.310	2.819	−8.472	2.717
*MDM4*	0.614	2.366	0.310	1.718	−5.151	1.647
***TP53***	−0.278	0.225	***0.003***	0.164	−0.828	−0.178
***TP53BP1***	−0.520	−0.052	***0.004***	0.161	−0.787	−0.150

(Significant p-values are marked in bold.)

^1^Mean mRNA z-scores; ^2^standard error; ^3^confidence of interval.

## DISCUSSION

Glioblastoma is one of the most assiduously studied cancer, yet the majority of patients are still resistant to the conventional therapies [1]. Thus, the identification of novel biomarkers to improve the management of GBM is an ongoing and challenging task [4]. Although PARP1 inhibition is a potential therapeutic target in GBM [5], its efficiency in the context of heterogeneity is unknown. Therefore, we performed an integrated bioinformatic analysis to evaluate *PARP1*'s genetic signature, and prognostic role regarding the molecular diversity of the tumour. Moreover, the correlations between PARP1 and three routinely used glioma markers detected by IHC were also tested in a clinical GBM cohort.

*PARP1* is overexpressed in a variety of cancers [7–9], including glioblastoma [23]. Increased *PARP1* expression has been reported in paediatric high-grade astrocytomas, medulloblastoma, and ependymoma [24, 25]. Furthermore, in GBM stem cells (GSCs), the combination of PARPi and TMZ may represent a valuable strategy to reverse the stem cells’ chemo-resistance. *Tentori et al*. found, that PARPi together with TMZ exerted synergistic anti-tumour effects in eight out of ten GSC lines. Moreover, the dose reduction of TMZ is associated with the sensitivity of each cell line to PARPi as single agent [26]. Recent bioinformatic analysis of TCGA datasets revealed that *PARP1* expression is restricted to higher grade tumours, and partially caused by genomic gain. Our findings underline that PARP1 is a marker candidate of higher-grade in astrocytomas, presumably because higher *PARP1* expression facilitates the repair of damaged DNA and, thereby, overcomes the genetic instability characteristics of tumour cells [27].

Given this complex heterogeneous nature of GBM, it is relevant to investigate PARP1's associations with key molecular markers [20, 21]. The importance of *ATRX*, *IDH1*, and *TP53* mutations in the early development and the progression of astrocytic glioma lineage is well-known [21]. These markers also have treatment and prognostic relevance [21] and their mutation status can distinguish astrocytomas from oligodendrogliomas, as well as secondary from primary GBM [20]. *PARP1* expression can occur in both IDH-wild type and mutated GBMs. Although there was no statistically proven association between PARP1 expression and IDH mutation status, a recent study reported that 2-hydroxyglutarate produced by mutated IDH induces PARP inhibitor sensitivity in patient-derived primary glioma cells and genetically matched tumour xenografts [28], which has therapeutic implications. On the other hand, higher *PARP1* expression levels were tightly associated with *ATRX* and *TP53* mutations. *ATRX* alterations occur in the vast majority of lower-grade astrocytomas and *IDH1*-mutated (secondary) GBMs [21]. Somatic mutations in *TP53* play important roles in gliomas, particularly in the tumorigenesis of lower grade astrocytomas and *IDH1*-mutated GBMs [4]. Considering that the inhibition of PARP1 enzyme is dose-dependent [29], our results indicate that PARP inhibitors could be more effective in *ATRX* and *TP53* mutated tumours, where *PARP1* levels are usually increased.

Although high-throughput technologies are widely used for diagnostic purposes, there is still a need to improve the IHC-based stratification of GBM with the integration of molecular data [30]. Consistent with a previous study [23], PARP1 IHC expression was observed in the majority of clinical GBM cases. To date, the IHC detection of key molecular markers is possible and highly informative: I) using *IDH1*^R132H^ mutation specific antibody [31]; II) most *ATRX* mutations result in undetectable *ATRX* expression by IHC [32]; and III) mutated p53 protein accumulates in the nucleus of tumour cells [33]. Furthermore, we have demonstrated that IHC expression of PARP1 has an inverse correlation with *ATRX*, and linear correlation with p53 staining. These observations suggest that PARP1 IHC expression along with p53 overexpression and *ATRX* loss can be promising predictive markers for PARPi in GBM.

This study investigates for the first time, to our knowledge, the genomic signature and prognostic significance of *PARP1* in GBM subtypes. We hereby present evidence, that *PARP1* can distinguish PN and CL from the other subtypes as increased *PARP1* expression was increased in these two subtypes (Figure 2C). Our data showed that high *PARP1* levels were associated with shorter survival in the CL group. It was previously presented that CL GBM is characterized by *EGFR* amplification and wild-type *TP53*, whereas PN is characterized by both *IDH1* and *TP53* mutations and *PDGFRA* amplification [22]. These results indicate that PARP1 and p53 are suitable markers to distinguish PN and CL subtypes, and have a prognostic relevance in GBM.

In our previous study, we demonstrated that p53 immunopositivity correlates with *TP53* mutational status, except for certain mutation types [33]. In GBM, we found that PARP1 IHC expression is correlated with p53 positive cases. In addition, *PAPR1* mRNA levels were higher in samples with *TP53* mutations. Importantly, the detection of *TP53* null mutations is not accurate by IHC due to the truncated protein [33]. Beside the increased *PARP1* levels in *TP53* mutated cases, there was no statistical difference between missense and null mutated samples.

Both PARP1 and p53 play important roles in maintaining genomic integrity [34, 35]. Several studies have shown that genomic instability is usually correlated with poor prognosis [17, 36]. It has been previously reported that high level of *PARP1* expression is often associated with poor overall survival in cancer [12]. Although there is a trend towards shorter survival of PARP1-high patients as compared to PARP1-low patients, no significant differences were observed when all GBMs were considered. Interestingly, we found a significantly shorter survival in PARP-high patients with wildtype *TP53* gene (Figure 6) – possibly because not only *TP53* mutations but also an impaired p53 pathway can facilitate tumour progression [37]. The p53 signalling pathway mediates several cellular processes including growth arrest, angiogenesis, apoptosis, and DNA repair [36]. It is widely accepted, that the progression and recurrence of glioblastoma is related to p53 pathway abnormalities in 87% of primary GBM [17]. To gain insights into the interaction between p53 and PARP1, we investigated and found that *PARP1* showed association with *CDKN2A* deletions and *MDM4* gain (both *CDKN2A* and *MDM4* are components of the p53 pathway) (Figure 7B). These two genetic alterations are frequent in GBM, and result in abnormal p53 signalling in tumour cells [17]. Furthermore, *PARP1* was negatively correlated with *CDKN1A*, which is a downstream member of the p53 cascade involved in the p53-mediated G1 arrest. This observation suggests that down-regulated *CDKN1A* cannot inhibit the Cyclin E-CDK2 complex in GBMs with higher *PARP1* levels. Consequently, it can result in an augmented cell-cycle activity and absence of apoptosis [38]. Furthermore, it has been reported, that low levels of 53BP1 could predict resistance to PARP inhibitors, because 53BP1 depletion reduces the cytotoxicity of PARP inhibitors [39]. Consistent with this notion, we found an overall low expression of *TP53BP1* in GBM with higher levels in high-PARP1 cases.

In conclusion, *PARP1* expression was increased in GBM at both mRNA and protein levels. To best of our knowledge, this is the first study to investigate the relevance of *PARP1* in the context of specific molecular markers and subtypes of GBM. We have demonstrated that increased *PARP1* levels show positive correlation with increasing tumour grades in gliomas Higher *PARP1* mRNA expression levels were associated with *ATRX* and *TP53* mutations. An IHC analysis in an independent clinical cohort also confirmed this relationship. Our results revealed that *PARP1* levels were increased in the PN and CL subtypes and correlated with shorter survival in CL GBMs. The observed subtype-specificity suggests that PARP1, together with p53, is not only a diagnostic marker to differentiate PN and CL subtypes, but also a predictive marker of shorter survival and poor therapy response in the CL subgroup. We also pointed out that there is a close interplay between PARP1 and the members of the p53 pathway in GBM. Our results support the therapeutic role of PARP inhibitors in high-grade gliomas with the caveat that molecular heterogeneity needs to be taken into account.

## MATERIALS AND METHODS

### TCGA datasets

Genetic alterations of *PARP1*, somatic mutation, CNA, and mRNA expression (z-score, RNA Seq V2 RSEM) data were collected from two TCGA cohorts: Glioblastoma Multiforme (TCGA, Provisional) and Brain Lower Grade Glioma (TCGA, Provisional) using the cBioPortal for Cancer Genomics (www.cbioportal.org). The relative expression of each gene and the gene expression distribution in a reference population were examined. The reference population was, either, all tumours that are diploid for the gene in question or, when available, have a normal adjacent tissue. The returned value indicates the number of standard deviations away from the mean of expression in the reference population (z-score) [40, 41]. In cBioPortal, copy numbers are computed using the GISTIC (Genomic Identification of Significant Targets in Cancer) algorithm, which identifies the putative copy number level as follows: I) high-level amplification, II) low-level gain, III) diploid, IV) heterozygous deletion, or V) homozygous deletion [40, 41].

Clinicopathological data for each patient including: age, sex, and survival time, was compiled from the TCGA portal (www.tcga-data.nci.nih.gov) and tabulated with genetic data. Cases for the analysis were selected according to the following criteria: I) only patients older than 18 years with available clinical data were included, considering that paediatric GBM represents a distinct molecular and genetic background (see reviews [42, 43]); II) oligodendrogliomas and mixed gliomas were omitted; III) tumours with complete genomic data (available somatic mutations, CNA, and mRNA expression information) were selected for the *PARP1* mRNA expression analysis. Altogether, 135 WHO grade IV glioblastoma and 96 lower grade astrocytomas (grade II & grade III) were assembled for the present analyses. The clinicopathological parameters, and key marker status of the cases are summarized in Table 2. GBM subtypes were accessible on 119 cases, with the following distribution: 57 ME, 43 CL, 16 PN, and 5 NE.

The relationships between *PARP1* mRNA expression and the overall patient survival were analysed by dividing the samples into PARP1-low and PARP1-high expression groups, based on median mRNA expression z-score in TCGA dataset.

### Patients with GBM

Samples were obtained from 60 patients (30 males and 30 female) diagnosed with GBM between 2006 and 2014 at the first author's affiliated institute. The mean age at diagnosis was 58.47 ± 9.03 years (range 30.21- 76.67 years). After surgical removal, sections were cut and stained with haematoxylin-eosin (H&E) from formalin-fixed and paraffin-embedded (FFPE) blocks. All the histopathological specimens were reviewed and diagnosed by a neuropathologist (TH) according to World Health Organization (WHO) criteria [19]. This research protocol was approved by the Institutional Review Board of the University of Debrecen.

### Immunohistochemistry

Immunohistochemical (IHC) analysis was carried out as previously described [44]. Briefly, the staining was performed on 4-μm-thick FFPE sections using the manufacturers’ protocols. At first, sections were deparaffinised in xylene and rehydrated in a graded series of ethanol. Heat-induced epitope retrieval was performed utilizing citrate buffer (pH 6.0). After blocking endogenous peroxidase activity, sections were incubated with primary antibodies (anti-p53, anti-ATRX, anti-IDH1, and anti-PARP1) for 6 hours at room temperature. Specifications of the antibodies are listed in Table 6. Visualization was achieved with SuperSensitive^™^ One-step Polymer-HRP Detection System on Leica Bond Max^™^ automated IHC stainer, employing 3,3′-diaminobenzidine (DAB), then followed by counterstaining with hematoxylin. Adequate positive and negative controls (with omission of the primary antibodies) were included in the immunohistochemical experiments.

**Table 6:** Specifications of the antibodies used for immunohistochemical staining

Antibodies	Catalogue numbers	Manufacturer	Dilutions
**anti-ATRX**	HPA001906	Sigma-Aldrich	1:1000
**anti-IDH1**	DIA-H09	Dianova	1:50
**anti-p53**	DO-7	Dako	1:700
**anti-PARP1**	ab6079	Abcam	1:500

Cases with ≥10 % stained cells were defined as positive for IDH1 and p53 [20]. A cut-off point of 10 % was considered for the evaluation of presence or absence of nuclear ATRX [32]. All FFPE sections were scored in a blinded manner by two independent observers (MC & RH). The expression of all IHC markers was determined semi-quantitatively. Ten randomly selected high-power fields (or the entire tumour if the sample was smaller) were examined in each neurosurgical specimen.

The PARP1 expression was evaluated based on the distribution of positive cells. The percentage of positivity was scored as follows: “0” (<5%, negative), “1” (5–25%, sporadic), “2” (25–50%, focal), or “3” (>50%, diffuse). For the statistical analysis, negative and sporadic staining was combined as low PARP1 staining and focal and diffuse nuclear staining as high PARP1 staining.

### Statistical and data analyses

Statistical analyses were performed using R software (http://www.r-project.org) and SPSS 21.0 software (SPSS Inc., IL, USA). Comparisons between groups were calculated with Pearson's Chi-square (χ2) test for categorical variables applying Yates' correction when required. The association between mRNA levels was measured using the Kendall's tau and Spearmann's correlation test. The difference in mRNA expression levels, between groups was calculated using a two-tailed Student's t-test. Kaplan–Meier method and Log-Rank (Mantel-Cox) test were used for overall survival measurements. Differences were significant when p<0.05.

Gene-gene interaction network to demonstrate p53 pathway and *PARP1* interaction was generated by the GeneMania Cytoscape 3.4.0 application. Physical, co-expression and gene-gene interactions were evaluated [45].

## SUPPLEMENTARY MATERIALS AND FIGURES


