# Supplementary material for: PARP1 expression and its correlation with survival is tumour molecular subtype dependent in glioblastoma
Source: Oncotarget. 2017 May 19;8(28):46348–62. doi: 10.18632/oncotarget.18013 (PMC5542272; doi:10.18632/oncotarget.18013)
Supplement: Supplementary file 1 [file oncotarget-08-46348-s001.pdf]

PARP1 expression and its correlation with survival is tumour molecular subtype dependent in glioblastoma

SUPPLEMENTARY MATERIALS

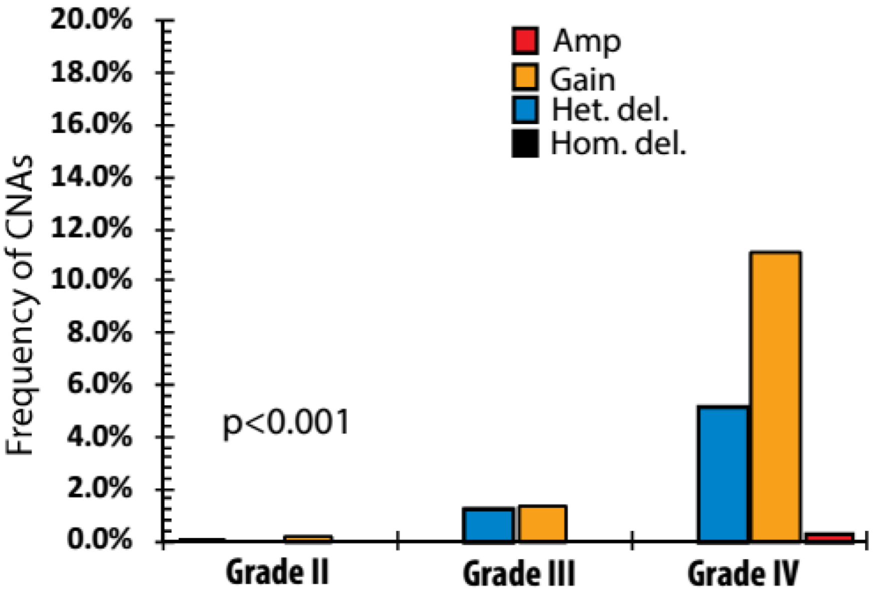

Supplementary Figure 1: Frequencies of PARP1 copy number alterations (CNAs) among glioma WHO grades according to the glioblastoma multiforme (WHO grade IV) & brain lower grade glioma (WHO grade II & III) TCGA datasets. $p<0.001$; $\chi 2$ test with Yates' correction.

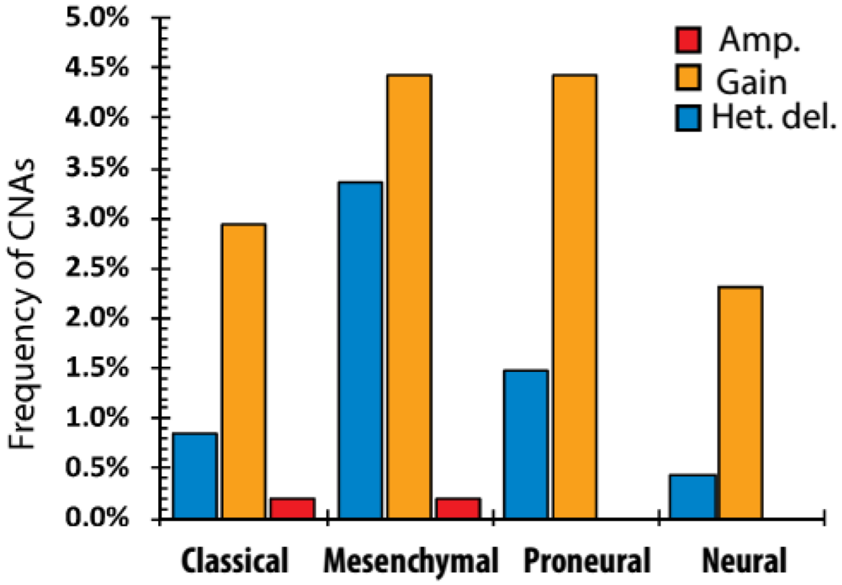

Supplementary Figure 2: Frequencies of PARP1 copy number alterations (CNAs) in glioblastoma subtypes. The TCGA glioblastoma multiforme, provisional dataset was used for the analysis. p = non-significant; χ2 test with Yates' correction.
